# A rare case of an indolent giant GH-secreting pituitary adenoma

**DOI:** 10.1210/jcemcr/luag138

**Published:** 2026-07-29

**Authors:** Shifa Shamim, Saira Furqan, Muhammad Nadeem Ahmad, Soman Prithiani

**Affiliations:** Section of Diabetes, Endocrinology and Metabolism, Medicine, The Aga Khan University, Karachi 74800, Pakistan; Section of Diabetes, Endocrinology and Metabolism, Medicine, The Aga Khan University, Karachi 74800, Pakistan; Department of Radiology, The Aga Khan University Hospital, Karachi 74800, Pakistan; Section of Diabetes, Endocrinology and Metabolism, Medicine, The Aga Khan University, Karachi 74800, Pakistan

**Keywords:** acromegaly, giant growth hormone secreting adenoma, pituitary adenoma

## Image legend

A 27-year-old woman presented with seizure, headache, and secondary amenorrhea after 6 years of acral and facial enlargement. She had not sought medical attention until the seizure. Hormonal workup revealed elevated insulin-like growth factor-1 of 704.90 ng/mL (704.9 µg/L) (normal range [NR]: 107.8-246.7 ng/mL [International System of Units (SI): 107.8-246.7 µg/L]), GH levels of 113 ng/mL (113 µg/L) (NR: 2.0-5.0 ng/mL [SI: 2.0-5.0 µg/L]), confirming acromegaly. Secondary hypothyroidism was found as TSH was 5.72 μIU/mL (5.72 mU/L) (NR: 0.4-4.2 µIU/mL [SI: 0.4-4.2 mU/L]) with free T4 of 0.43 ng/dL (5.4 pmol/L) (NR: 0.92-1.68 ng/dL [SI: 10-36 pmol/L]). Two values of morning cortisol were <10 μg/dL (<276 nmol/L) (8 Am NR: 4.82-19.5 μg/dL [SI: 133-538 nmol/L]). Prolactin and gonadotropin levels were normal. Magnetic resonance imaging of the brain (Fig. 1A and 1B) revealed a large, multilobulated, heterogeneously enhancing pituitary adenoma measuring 94.8 × 73 × 79.3 mm. Adrenal insufficiency and secondary hypothyroidism were managed with daily hydrocortisone (10 mg) and levothyroxine (100 mcg), respectively. She underwent transcranial resection of the adenoma. Postoperatively, she developed transient arginine vasopressin deficiency. Giant pituitary adenomas comprise 6% to 10% of pituitary tumors [1]. Delaying medical evaluation until seizures occurred, despite significant tumor burden, demonstrates why clinicians should maintain a high index of suspicion for sellar lesions when symptoms seem mild or nonspecific.

**Figure:**
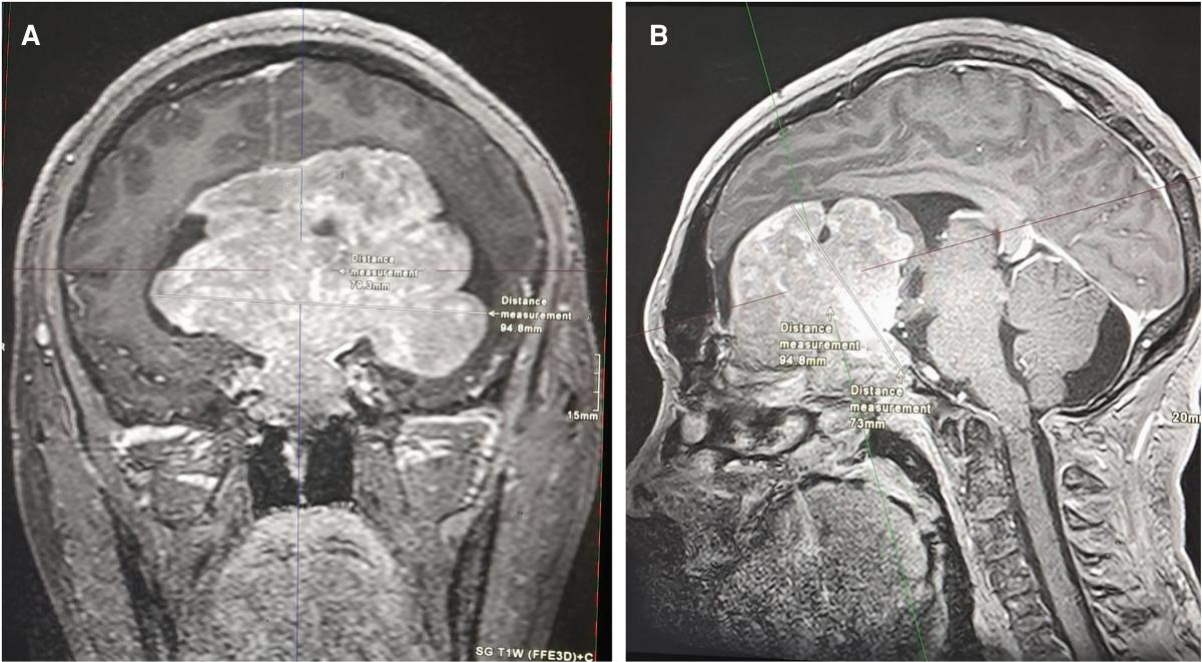


## Contributors

All authors made individual contributions to authorship. S.S, S.F, and S.P were involved in the diagnosis and management of the patient. M.N.A was involved in interpreting and providing the radiological images. S.S was involved in the manuscript preparation. All authors reviewed and approved the final draft.

## Abbreviations

NR: normal range
SI: International System of Units
